# Dental Tissue Density in Healthy Children Based on Radiological Data: Retrospective Analysis

**DOI:** 10.2196/56759

**Published:** 2024-06-20

**Authors:** Aleksey Reshetnikov, Natalia Shaikhattarova, Margarita Mazurok, Nadezhda Kasatkina

**Affiliations:** 1 Department of Oral Surgery, Resto Dental Clinic Ltd, Izhevsk, Russian Federation; 2 Department of Dentistry, Lobachevsky State University, Nizhny Novgorod, Russian Federation

**Keywords:** density, teeth, tooth, dental, dentist, dentists, dentistry, oral, tissue, enamel, dentin, Hounsfield, pathology, pathological, radiology, radiological, image, images, imaging, teeth density, Hounsfield unit, diagnostic imaging

## Abstract

**Background:**

Information about the range of Hounsfield values for healthy teeth tissues could become an additional tool in assessing dental health and could be used, among other data, for subsequent machine learning.

**Objective:**

The purpose of our study was to determine dental tissue densities in Hounsfield units (HU).

**Methods:**

The total sample included 36 healthy children (n=21, 58% girls and n=15, 42% boys) aged 10-11 years at the time of the study. The densities of 320 teeth tissues were analyzed. Data were expressed as means and SDs. The significance was determined using the Student (1-tailed) *t* test. The statistical significance was set at *P*<.05.

**Results:**

The densities of 320 teeth tissues were analyzed: 72 (22.5%) first permanent molars, 72 (22.5%) permanent central incisors, 27 (8.4%) second primary molars, 40 (12.5%) tooth germs of second premolars, 37 (11.6%) second premolars, 9 (2.8%) second permanent molars, and 63 (19.7%) tooth germs of second permanent molars. The analysis of the data showed that tissues of healthy teeth in children have different density ranges: enamel, from mean 2954.69 (SD 223.77) HU to mean 2071.00 (SD 222.86) HU; dentin, from mean 1899.23 (SD 145.94) HU to mean 1323.10 (SD 201.67) HU; and pulp, from mean 420.29 (SD 196.47) HU to mean 183.63 (SD 97.59) HU. The tissues (enamel and dentin) of permanent central incisors in the mandible and maxilla had the highest mean densities. No gender differences concerning the density of dental tissues were reliably identified.

**Conclusions:**

The evaluation of Hounsfield values for dental tissues can be used as an objective method for assessing their densities. If the determined densities of the enamel, dentin, and pulp of the tooth do not correspond to the range of values for healthy tooth tissues, then it may indicate a pathology.

## Introduction

Healthy hard and soft dental tissues determine the quality of human life. Nowadays, there are various methods of clinical, laboratory, and instrumental studies that allow us not only to assess the initial condition of hard and soft tooth tissues but also to evaluate their change during therapeutic and preventive procedures [1,2]. Dynamic monitoring of dental tissue condition is required in trauma, after transplantation, and during therapeutic and preventive procedures [3-5]. It is especially important in children with metabolic diseases, genetic abnormalities, and special needs [6-8]. The emergence of innovative diagnostic methods provides dentists with new opportunities to assess dental health, especially in the early stages of pathological changes that are not visible to the eye. Recently, cone-beam computed tomography (CBCT) has been widely used in dentistry [9]. Unlike traditional orthopantomograms, CBCT allows the clinician to analyze tissue density using Hounsfield units (HU) [10,11]. Information about the range of Hounsfield values for healthy teeth tissues could become an additional tool in assessing dental health, age estimation [12], and anatomy [13] and could be used, among other data, for subsequent machine learning [14]. The results of earlier studies do not provide convincing data on the range of Hounsfield values for healthy dental tissues in children of a certain age group [15,16]. Our study is aimed at establishing the Hounsfield values of dental tissue density in children in the same age group.

## Methods

### Ethical Considerations

The study was conducted at Resto Dental Clinic Ltd, Izhevsk, Russia, from January 2021 to January 2023. The study protocol complied with the principles outlined in the Declaration of Helsinki of the World Health Organization and was approved by the Ethics Committees at Resto Dental Clinic Ltd (protocol 07; December 22, 2020). Informed consent was obtained from the parents or legal guardians of all children in the study.

### Participants

The study included 36 children aged 10‐11 years of both genders. The criteria for including children in the study were (1) the presence of medical indications for CBCT (malocclusion and dental structural anomalies in the primary and permanent dentition, dental trauma, or anomalies in dental position), (2) aged over 10 years, (3) consent to the study, and (4) the absence of genetic anomalies and concomitant diseases.

### Procedures

**Figure 1.**
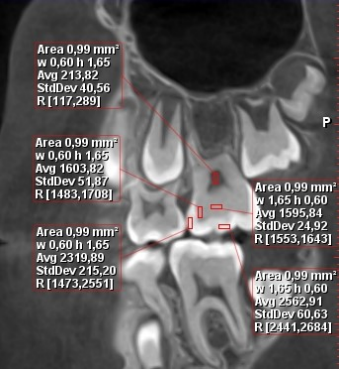
Measuring the radiodensity (in Hounsfield units) of a first permanent molar of a boy (10 years old) under cone-beam computed tomography. H: height; W: width.

This study was not a randomized controlled trial and was therefore not registered at ClinicalTrials.gov. Before CBCT, all participants underwent a clinical study with a visual-tactile method. CBCT studies were performed using a PlanmecaProMax 3D tomograph (Planmeca Oy) with scanning parameters of 88 kV, 5 mA, and 15 seconds. Only 1 expert clinician performed the measurements. PlanmecaRomexis 5.2.R 24.10.18 software (Planmeca Oy) was used to analyze the data obtained. The average dental tissue density was determined over an area of 1 mm^2^. Teeth tissues of the upper and lower jaws that were selected for the study included first permanent molars, permanent central incisors, second primary molars, tooth germs of second premolars, second premolars, second permanent molars, and tooth germs of second permanent molars. Teeth enamel and dentin densities were measured in HU on the incisor or occlusal surface (enamel 1 and dentin 1) and proximal surface (enamel 2 and dentin 2). Pulp density was measured in its central area (Figure 1).

### Data Analysis

Data were expressed as means and SDs. The significance was determined using the Student (1-tailed) *t* test. The statistical significance was set at *P*<.05.

## Results

### Baseline Characteristics

The total sample consisted of 36 healthy children (n=21, 58% girls and n=15, 42% boys) aged 10‐11 years at the time of the study. The densities of 320 teeth tissues were analyzed: 72 (22.5%) first permanent molars, 72 (22.5%) permanent central incisors, 27 (8.4%) second primary molars, 40 (12.5%) tooth germs of second premolars, 37 (11.6%) second premolars, 9 (2.8%) second permanent molars, and 63 (19.7%) tooth germs of second permanent molars.

### Dental Tissue Densities

The analysis of the data showed that tissues of healthy teeth in children have different density ranges: enamel, from mean 2954.69 (SD 223.77) HU to mean 2071.00 (SD 222.86) HU; dentin, from mean 1899.23 (SD 145.94) HU to mean 1323.10 (SD 201.67) HU; and pulp, from mean 420.29 (SD 196.47) HU to mean 183.63 (SD 97.59) HU. The statistical analysis did not reveal any significant relationships between Hounsfield values and demographic data (gender). Therefore, the densities of the tissues of the maxilla and mandible teeth were compared. Detailed data are presented in Table 1.

The tissues (enamel and dentin) of permanent central incisors in the mandible and maxilla had the highest mean densities. The enamel and dentin densities of the second primary molars were significantly lower than those for second permanent molars and tooth germs of second permanent molars (all *P*<.05).

**Table 1.** Dental tissue densities of healthy children in Hounsfield units.

Teeth tissues and jaw		Enamel 1	Enamel 2	Dentin 1	Dentin 2
**First permanent molars**					
	Maxilla (n=36), mean (SD)	2426.28 (168.41)	2358.81 (219.60)	1561.17 (143.59)	1584.11 (137.17)
	Mandible (n=36), mean (SD)	2414.53 (194.85)	2336.39 (171.98)	1537.50 (150.25)	1487.19 (189.15)
	*t* test (*df*)^a^	0.2699 (70)	0.4756 (70)	0.6738 (70)	2.4543 (70)
	*t* critical value^b^	2.0003	2.0003	2.0003	2.0003
**Permanent central incisors**					
	Maxilla (n=36), mean (SD)	2954.69 (223.77)	2592.54 (186.54)	1796.40 (163.39)	1791.91 (127.94)
	Mandible (n=36), mean (SD)	2984.20 (223.44)	2552.37 (186.85)	1899.23 (145.94)	1871.69 (98.81)
	*t* test (*df*)	0.5521 (70)	0.9001 (70)	2.7769 (70)	2.9202 (70)
	*t* critical value	2.0003	2.0003	2.0003	2.0003
**Second primary molars**					
	Maxilla (n=16), mean (SD)	2141.75 (246.70)	2228.53 (160.24)	1428.06 (203.41)	1413.21 (145.79)
	Mandible (n=11), mean (SD)	2227.09 (115.66)	2071.00 (222.86)	1323.10 (201.67)	1434.50 (144.22)
	*t* test (*df*)	1.2045 (25)	1.9976 (25)	1.2867 (25)	0.355 (25)
	*t* critical value	2.0595	2.0639	2.0639	2.0739
**Tooth germs of second premolars**					
	Maxilla (n=21), mean (SD)	2449.71 (181.11)	2509.62 (221.56)	1576.48 (126.62)	1649.71 (128.85)
	Mandible (n=19), mean (SD)	2583.68 (134.75)	2611.32 (181.89)	1666.42 (138.10)	1695.74 (108.76)
	*t* test (*df*)	2.6698 (38)	1.5923 (38)	2.1394 (38)	1.2245 (38)
	*t* critical value	2.0211	2.0211	2.0211	2.0211
**Second premolars**					
	Maxilla (n=20), mean (SD)	2220.58 (190.65)	2301.32 (193.38)	1417.30 (119.57)	1507.85 (171.50)
	Mandible (n=17), mean (SD)	2336.94 (218.79)	2348.18 (103.87)	1337.00 (170.81)	1375.18 (126.17)
	*t* test (*df*)	1.7094 (35)	0.9365 (35)	1.6285 (35)	2.7042 (35)
	*t* critical value	2.0211	2.0211	2.0211	2.0211
**Second permanent molars**					
	Maxilla (n=4), mean (SD)	2350.00 (49.02)	2403.50 (101.93)	1569.00 (88.75)	1523.25 (91.31)
	Mandible (n=5), mean (SD)	2293.40 (131.28)	2174.40 (145.79)	1443.00 (70.81)	1327.60 (121.99)
	*t* test (*df*)	0.8897 (7)	2.7734 (7)	2.3111 (7)	2.7502 (7)
	*t* critical value	2.3646	2.3646	2.3646	2.3646
**Tooth germs of second permanent molars**					
	Maxilla (n=32), mean (SD)	2359.03 (169.39)	2403.16 (209.89)	1527.03 (121.39)	1519.66 (105.13)
	Mandible (n=31), mean (SD)	2356.52 (148.88)	2499.97 (178.51)	1527.81 (128.91)	1554.37 (120.88)
	*t* test (*df*)	0.0625 (61)	1.9741 (61)	0.0247 (61)	1.2031 (61)
	*t* critical value	2.0423	2.0423	2.0423	2.0423

a The differences are significant when *t* test value>*t* critical value at *P*=.05.

b *t* critical value at *P*=.05.

## Discussion

### Principal Findings

The densities of dental tissues are an independent sign of their health. With the development of computed tomography and software, clinicians acquired an additional tool for analyzing the density of dental tissues [11]. The determination of Hounsfield values of dental tissues using CBCT can be used as an objective method for assessing their densities in people of different age groups. We obtained the measurements of tissue densities of healthy teeth in children aged 10‐11 years.

Previous studies of extracted teeth using microcomputed tomography showed uneven distribution of enamel and dentin densities in different areas of the tooth [14]. Yavuz et al [15] confirmed this pattern in a mixed-age population using CBCT in their study. However, the densities of enamel and dentin in their study were lower than the average values obtained during our study. One of the reasons justifying this difference may be the fact that our study included children in the same age group, which may justify further studies on the dental tissue density in a population of children and adults of certain age groups. The obtained densities for the tissues of teeth germs indicate that they correspond to the densities of permanent teeth and exceed similar indicators of the densities of primary teeth tissues.

We believe that further research on the density range for healthy and pathologically altered dental tissues, as well as study standardization, can help clinicians improve the accuracy of screening and optimize subsequent monitoring of the effectiveness of preventive and therapeutic procedures in the future. This study is an attempt to establish the range of Hounsfield values for healthy maxillary and mandibular dental tissues in children of a certain age group. The data obtained revealed the densities for enamel, dentin, and pulp for primary and permanent teeth and germs of primary teeth. Differences in the densities of specific teeth were also revealed; in particular, it was found that the enamel of the incisors had the highest density, significantly exceeding the densities of the molars. Further research on the densities of dental tissues in normal and pathological conditions seems promising, in particular for machine learning [14,17].

### Limitations

A limitation of our study was that measurements were carried out by only 1 expert clinician, which eliminates an assessment of interobserver variability. The study was conducted in a population of children in the same age group. In addition, not all maxillary and mandibular teeth were included in the study. This study only obtained Hounsfield values of dental structures from 1 particularly used CBCT machine. Further studies on a larger population may be useful to improve the information content of the data.

### Conclusions

The evaluation of Hounsfield values of dental tissues can be used as an objective method for assessing their densities. If the determined densities of the enamel, dentin, and pulp of the tooth do not correspond to the range of values for healthy tooth tissues, then it may indicate a pathology.

## Abbreviations

CBCT: cone-beam computed tomography
HU: Hounsfield units
